# Supplementary material for: Unveiling the Bioactive Potential of the Invasive Jellyfish Phyllorhiza punctata Through Integrative Transcriptomic and Proteomic Analyses
Source: Biomolecules. 2025 Aug 4;15(8):1121. doi: 10.3390/biom15081121 (PMC12383608; doi:10.3390/biom15081121)
Supplement: Supplementary file 1 [file biomolecules-15-01121-s001.zip › Suplementary Material/FileS3/PEPFOLD4/XP_065065372_8460/clusters.html]

 PEP-FOLD clusters


Download archive of all models  here   

|  |  |  |
| --- | --- | --- |
| # | file | sOPEP |
| # cluster 1 - Size: 98 |  |  |
|  | XP\_065065372\_8460-model1 | -14.8172 |
|  | XP\_065065372\_8460-model1.1 | -14.7214 |
|  | XP\_065065372\_8460-model1.2 | -14.7214 |
|  | XP\_065065372\_8460-model1.3 | -14.7214 |
|  | XP\_065065372\_8460-model1.4 | -14.7068 |
|  | XP\_065065372\_8460-model1.5 | -14.7068 |
|  | XP\_065065372\_8460-model1.6 | -14.6883 |
|  | XP\_065065372\_8460-model1.7 | -14.6808 |
|  | XP\_065065372\_8460-model1.8 | -14.6798 |
|  | XP\_065065372\_8460-model1.9 | -14.6798 |
|  | XP\_065065372\_8460-model1.10 | -14.6798 |
|  | XP\_065065372\_8460-model1.11 | -14.6701 |
|  | XP\_065065372\_8460-model1.12 | -14.6701 |
|  | XP\_065065372\_8460-model1.13 | -14.6701 |
|  | XP\_065065372\_8460-model1.14 | -14.6658 |
|  | XP\_065065372\_8460-model1.15 | -14.6613 |
|  | XP\_065065372\_8460-model1.16 | -14.6334 |
|  | XP\_065065372\_8460-model1.17 | -14.6208 |
|  | XP\_065065372\_8460-model1.18 | -14.6208 |
|  | XP\_065065372\_8460-model1.19 | -14.6198 |
|  | XP\_065065372\_8460-model1.20 | -14.6198 |
|  | XP\_065065372\_8460-model1.21 | -14.6198 |
|  | XP\_065065372\_8460-model1.22 | -14.6198 |
|  | XP\_065065372\_8460-model1.23 | -14.6198 |
|  | XP\_065065372\_8460-model1.24 | -14.6198 |
|  | XP\_065065372\_8460-model1.25 | -14.6198 |
|  | XP\_065065372\_8460-model1.26 | -14.6198 |
|  | XP\_065065372\_8460-model1.27 | -14.6156 |
|  | XP\_065065372\_8460-model1.28 | -14.6156 |
|  | XP\_065065372\_8460-model1.29 | -14.6113 |
|  | XP\_065065372\_8460-model1.30 | -14.6094 |
|  | XP\_065065372\_8460-model1.31 | -14.6094 |
|  | XP\_065065372\_8460-model1.32 | -14.5952 |
|  | XP\_065065372\_8460-model1.33 | -14.5952 |
|  | XP\_065065372\_8460-model1.34 | -14.5952 |
|  | XP\_065065372\_8460-model1.35 | -14.5946 |
|  | XP\_065065372\_8460-model1.36 | -14.5946 |
|  | XP\_065065372\_8460-model1.37 | -14.5932 |
|  | XP\_065065372\_8460-model1.38 | -14.5932 |
|  | XP\_065065372\_8460-model1.39 | -14.59 |
|  | XP\_065065372\_8460-model1.40 | -14.5874 |
|  | XP\_065065372\_8460-model1.41 | -14.5874 |
|  | XP\_065065372\_8460-model1.42 | -14.587 |
|  | XP\_065065372\_8460-model1.43 | -14.5866 |
|  | XP\_065065372\_8460-model1.44 | -14.5866 |
|  | XP\_065065372\_8460-model1.45 | -14.5866 |
|  | XP\_065065372\_8460-model1.46 | -14.5827 |
|  | XP\_065065372\_8460-model1.47 | -14.5827 |
|  | XP\_065065372\_8460-model1.48 | -14.5827 |
|  | XP\_065065372\_8460-model1.49 | -14.5805 |
|  | XP\_065065372\_8460-model1.50 | -14.5713 |
|  | XP\_065065372\_8460-model1.51 | -14.5713 |
|  | XP\_065065372\_8460-model1.52 | -14.5626 |
|  | XP\_065065372\_8460-model1.53 | -14.5574 |
|  | XP\_065065372\_8460-model1.54 | -14.5566 |
|  | XP\_065065372\_8460-model1.55 | -14.5566 |
|  | XP\_065065372\_8460-model1.56 | -14.5566 |
|  | XP\_065065372\_8460-model1.57 | -14.5563 |
|  | XP\_065065372\_8460-model1.58 | -14.5563 |
|  | XP\_065065372\_8460-model1.59 | -14.5563 |
|  | XP\_065065372\_8460-model1.60 | -14.5523 |
|  | XP\_065065372\_8460-model1.61 | -14.5523 |
|  | XP\_065065372\_8460-model1.62 | -14.5523 |
|  | XP\_065065372\_8460-model1.63 | -14.5443 |
|  | XP\_065065372\_8460-model1.64 | -14.5443 |
|  | XP\_065065372\_8460-model1.65 | -14.544 |
|  | XP\_065065372\_8460-model1.66 | -14.544 |
|  | XP\_065065372\_8460-model1.67 | -14.5434 |
|  | XP\_065065372\_8460-model1.68 | -14.5434 |
|  | XP\_065065372\_8460-model1.69 | -14.5434 |
|  | XP\_065065372\_8460-model1.70 | -14.5434 |
|  | XP\_065065372\_8460-model1.71 | -14.5434 |
|  | XP\_065065372\_8460-model1.72 | -14.5403 |
|  | XP\_065065372\_8460-model1.73 | -14.5403 |
|  | XP\_065065372\_8460-model1.74 | -14.5403 |
|  | XP\_065065372\_8460-model1.75 | -14.5403 |
|  | XP\_065065372\_8460-model1.76 | -14.5385 |
|  | XP\_065065372\_8460-model1.77 | -14.53 |
|  | XP\_065065372\_8460-model1.78 | -14.5226 |
|  | XP\_065065372\_8460-model1.79 | -14.5226 |
|  | XP\_065065372\_8460-model1.80 | -14.5226 |
|  | XP\_065065372\_8460-model1.81 | -14.5226 |
|  | XP\_065065372\_8460-model1.82 | -14.5226 |
|  | XP\_065065372\_8460-model1.83 | -14.5181 |
|  | XP\_065065372\_8460-model1.84 | -14.5181 |
|  | XP\_065065372\_8460-model1.85 | -14.5181 |
|  | XP\_065065372\_8460-model1.86 | -14.5162 |
|  | XP\_065065372\_8460-model1.87 | -14.5162 |
|  | XP\_065065372\_8460-model1.88 | -14.5078 |
|  | XP\_065065372\_8460-model1.89 | -14.5078 |
|  | XP\_065065372\_8460-model1.90 | -14.5016 |
|  | XP\_065065372\_8460-model1.91 | -14.498 |
|  | XP\_065065372\_8460-model1.92 | -14.4931 |
|  | XP\_065065372\_8460-model1.93 | -14.4931 |
|  | XP\_065065372\_8460-model1.94 | -14.4671 |
|  | XP\_065065372\_8460-model1.95 | -14.4671 |
|  | XP\_065065372\_8460-model1.96 | -14.4549 |
|  | XP\_065065372\_8460-model1.97 | -14.4011 |
| # cluster 2 - Size: 2 |  |  |
|  | XP\_065065372\_8460-model2 | -9.58998 |
|  | XP\_065065372\_8460-model2.1 | -9.46071 |
|  |  |  |
